# From Sperm Motility to Sperm-Borne microRNA Signatures: New Approaches to Predict Male Fertility Potential

**DOI:** 10.3389/fcell.2020.00791

**Published:** 2020-08-21

**Authors:** Maíra Bianchi Rodrigues Alves, Eneiva Carla Carvalho Celeghini, Clémence Belleannée

**Affiliations:** ^1^CHU de Québec Research Center (CHUL), Department of Obstetrics, Gynecology and Reproduction, Faculty of Medicine, Université Laval, Quebec City, QC, Canada; ^2^Department of Animal Reproduction, Universidade de São Paulo, Pirassununga, Brazil

**Keywords:** infertility, diagnosis, spermatozoa, semen, intrinsic factors, ncRNAs, human, cattle

## Abstract

In addition to the paternal genome, spermatozoa carry several intrinsic factors, including organelles (e.g., centrioles and mitochondria) and molecules (e.g., proteins and RNAs), which are involved in important steps of reproductive biology such as spermatogenesis, sperm maturation, oocyte fertilization and embryo development. These factors constitute potential biomarkers of “viable sperm” and male fertility status and may become major assets for diagnosing instances of idiopathic male infertility in both humans and livestock animals. A better understanding of the mechanism of action of these sperm intrinsic factors in the regulation of reproductive and developmental processes still presents a major challenge that must be addressed. This review assembles the main data regarding morpho-functional and intrinsic sperm features that are associated with male infertility, with a particular focus on microRNA (miRNA) molecules.

## Introduction

Male fertility potential relies on several physical, endocrine, and genetic factors, which underlie the production of fully functional spermatozoa as well as their successful arrival at the site of fertilization. Impairment of these male reproductive functions accounts for half of the infertility issues that couples face during their reproductive age (Agarwal et al., 2015; Zegers-Hochschild et al., 2017). While male infertility diagnosis is mainly based on physical characteristics, hormonal analysis, and use of the traditional spermogram, its etiology remains unexplained in more than 30% of patients (Irvine, 1998; Agarwal et al., 2015). Thus, the design of novel customized diagnostic tools to fully assess male fertility potential and male infertility etiology is a necessity. Furthermore, an improved method to assess male fertility potential would be a major asset to the livestock industry, since this parameter constitutes a serious economic challenge in this field. Research performed on sperm samples from humans, livestock animals and rodents recently questioned the definition of the sperm cell as a simple carrier of the paternal genome. From this, it now appears that the spermatozoon is a well-differentiated cell that carries a widely diverse signature of specific organelles and molecules, that could vary according to male fertility potential, and be transferred to the oocyte at the time of fertilization. This contemporary view of the sperm cell opens potential new avenues concerning the development of a novel generation of diagnostic tools. In this review, we will delineate structural, morpho-functional and intrinsic sperm features, and comment on their contribution to the post-fertilization processes. The detection of sperm microRNAs (miRNAs) as a predictor of male fertility potential will be discussed with regard to its possible applications in both clinic and industry.

## “Healthy Sperm” Concept: Structural, Morpho-Functional and Intrinsic Features

**FIGURE 1:**
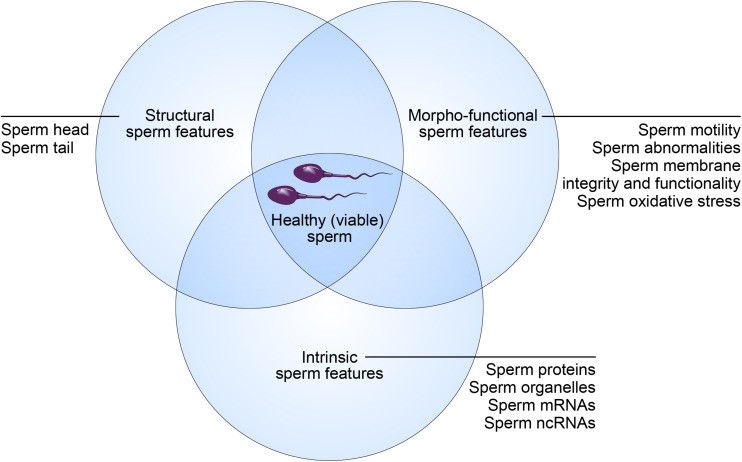
Concept of “healthy (viable) sperm.” Male fertility potential relies on structural, morpho-functional, and intrinsic sperm features that shape equally the concept of “healthy (viable) sperm.”

While sperm quality is a key determinant of male fertility potential, the discrimination between a high- and low-quality sperm sample is challenging. Indeed, this relies on a broad spectrum of sperm features (Amann and Hammerstedt, 1993). According to this concept, “healthy spermatozoa” also referred to as “viable spermatozoa” should possess the ability to reach the fertilization site, bind to and fertilize the oocytes, and properly contribute to initiation of early embryo development (Krawetz, 2005). These abilities are strictly dependent on specific structural, morpho-functional, and intrinsic features (Figure 1).

### Structural Sperm Features

**FIGURE 2:**
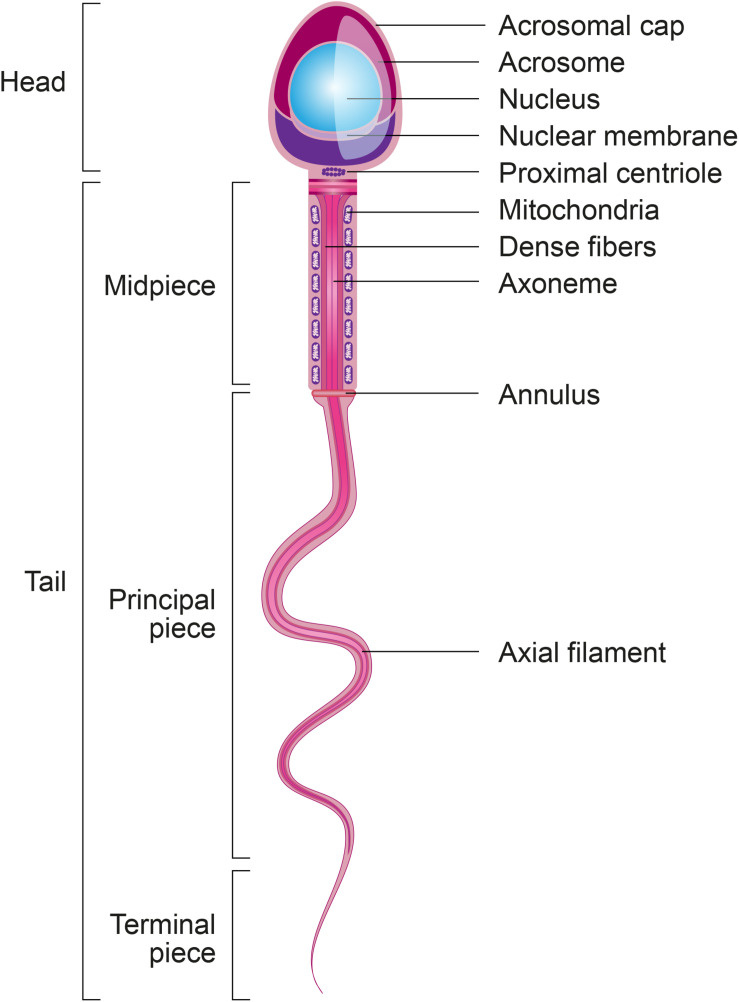
Structural sperm features. Spermatozoa are composed of two main parts: head and tail (or flagellum). The sperm head is constituted basically by the acrosome and nucleus. The sperm tail includes: the neck that contains mainly the proximal centriole; the midpiece which is composed by mitochondria, outer dense fibers (ODF) and axoneme; principal piece containing the fibrous sheath and axoneme; and terminal piece.

Spermatozoa are the most differentiated cells of the organism and display particular features related to their main function: the delivery of paternal DNA to the oocyte (Krawetz, 2005). In this regard, spermatozoa are composed of two main parts: a head to carry and protect the genome, and a tail or flagellum to propel the cell to the site of fertilization (Figure 2).

Within the **sperm head** is a limited quantity of cytoplasm, highly condensed DNA, and a well-delimited acrosome. A substantial portion of the cytoplasm is lost during the final steps of spermatogenesis, specifically during spermiogenesis, the process during which round spermatids differentiate into elongated spermatids and then spermatozoa (Gadea et al., 2013). The remaining cytoplasm from intercellular bridges, called the cytoplasmic droplet, is lost during sperm transit through the epididymis (Cooper and Yeung, 2003). During spermiogenesis, the sequential replacement of histones by transitional proteins and then by protamines within sperm chromatin triggers genome condensation (Zini and Agarwal, 2011; Gadea et al., 2013). The acrosome, located at the top of the head, contains specific enzymes that promote specific functions trailing sperm capacitation and acrosome reaction: (1) exposure of acrosome zona pellucida binding proteins during sperm capacitation; (2) sperm ability to cross cumulus cells that surrounds the oocytes, (3) sperm binding to zona pellucida and acrosome reaction, and (4) migration of IZUMO1 protein from the outer acrosomal membrane to the equatorial segment of sperm surface to ensure its binding to JUNO receptor on the oocyte (Satouh et al., 2012; Bianchi et al., 2014). Since acrosome reacted sperm retain the ability to penetrate the zona pellucida, the previous paradigm supporting the role of enzymes released during acrosome reaction in the digestion of the zona pellucida has been revisited in different species (Hirohashi and Yanagimachi, 2018).

The **sperm tail** (or flagellum) includes the neck, midpiece, principal piece and terminal piece. The sperm centrioles are important to early embryo development and are localized in the sperm neck (Avidor-Reiss and Fishman, 2019). The axoneme, located internally along the entire flagellum, is composed of nine peripheral doublets and two central single microtubules (9 + 2 structure) integrated by the intraflagellar transport (IFT) system. Surrounding the axoneme, there is the outer dense fibers (ODF) and mitochondria in the sperm midpiece and the fibrous sheath (FS), formed by nine bundles of fibers of different lengths, in the principal piece (Inaba, 2003). Depending on species, approximately 22–75 mitochondria are present in the midpiece to produce enough energy necessary for a spermatozoon to transit along the female reproductive tract and to reach the fertilization site in the oviduct (Song and Lewis, 2008). All of these sperm structural characteristics are essential to ensure the ability of spermatozoa to cross the muco-cervical and uterine barriers and reach the oviduct, where they bind to and penetrate the oocyte to deliver their DNA content.

### Morpho-Functional Sperm Features

**FIGURE 3:**
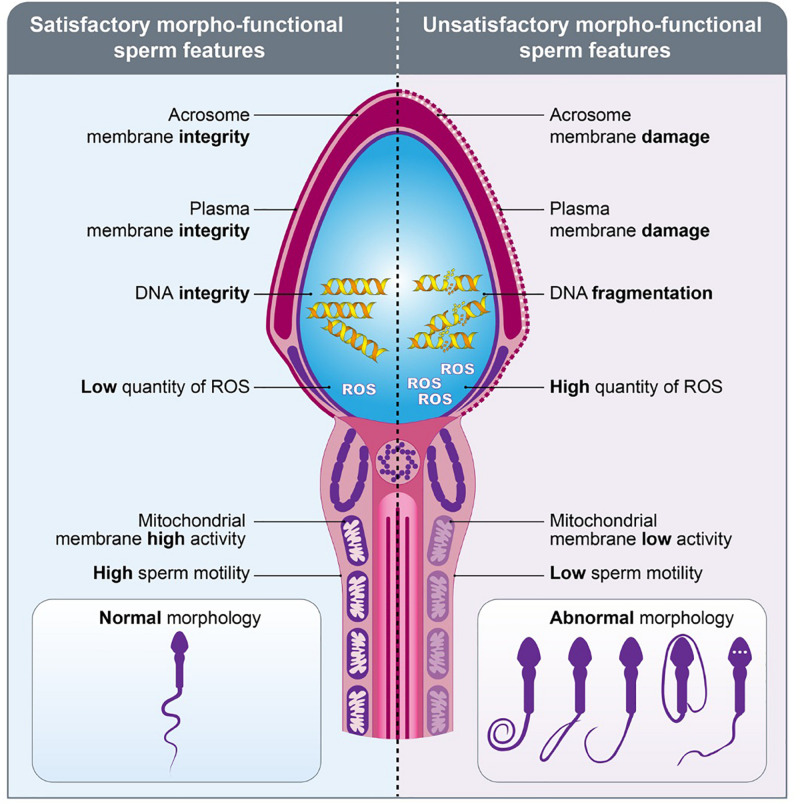
Morpho-functional sperm features. Schematic figure representing the spermatozoa with satisfactory **(left)** and unsatisfactory **(right)** morpho-functional features. Sperm acrosome membrane integrity, sperm plasma membrane integrity, sperm DNA integrity, low quantity of ROS, sperm mitochondrial membrane high activity, high sperm motility and normal sperm morphology characterize the satisfactory morpho-functional sperm features. Sperm acrosome membrane damage, sperm plasma membrane damage, sperm DNA fragmentation, high quantity of ROS, sperm mitochondrial membrane low activity, low sperm motility and abnormal sperm morphology characterize the unsatisfactory morpho-functional sperm features.

Morpho-functional sperm attributes are capable of modulating male fertility potential and are tightly related to the structural sperm features. Parameters such as **sperm motility/kinetics, morphological abnormalities, integrity of plasma and acrosome membranes, mitochondrial activity production of reactive oxygen species (ROS), DNA fragmentation** and **capacitation status** are included in this group and usually are associated with male fertility (Table 1 and Figure 3). Thus, assessment of these parameters is essential to determine male fertility potential: evaluation of a higher number of morpho-functional attributes establishes the highest relationship with male fertility (Amann and Hammerstedt, 1993). A high proportion of **morphological abnormalities** – referred to as teratozoospermia – including, e.g., spermatozoa with large, small or piriform heads as well as coiled-tails, is associated with reproductive dysfunctions (Gillan et al., 2008). This common cause of male infertility is routinely assessed by light microscopic analysis of semen in fertility clinics.

**TABLE 1:** Correlation between sperm morpho-functional attributes and male fertility.

**Sperm morpho-functional attribute**	**Definition**	**Required equipment**	**Correlation with fertility**	**References**
Sperm subjective motility	Maximum percentage of sperm cells with movement	Light field optical microscope, preferably with phase contrast	*r* = 0.53 (*P* < 0.01) (^1^60 to 90-day non-return)	Correa et al., 1997
			*r* = 0.67 (*P* = 0.03) (^1^56-day non-return)	Gillan et al., 2008
Computer assisted sperm analysis (CASA)	Percentage of sperm with motility and measurement of different sperm kinetic features	Light field optical microscope, with phase contrast coupled to a computer	WOB^2^: *r* = 0.57 (*P* < 0.05) (^1^56-day non-return)	Morrell et al., 2018
			*r* = 0.67 (*P* = 0.03) (^1^56-day non-return)	Gillan et al., 2008
			VSL^3^: *r* = −0.34 (*P* < 0.05) (^1^56-day non-return)	Sellem et al., 2015
			ALH^4^ and progressive motility presented *r*^2^ of 0.68	Farrell et al., 1998
			ALH^4^, BCF^5^, LIN^6^, VAP^7^ and VSL^3^ presented *r*^2^ of 0.98	Farrell et al., 1998
Sperm morphological abnormalities	Percentage of cells that present with form defects, often called abnormal cell percentage (Blom, 1973)	Light field optical microscope preferably with differential interference contrast	*r* = −0.59 (*P* = 0.01) (^1^60 to 90-day non-return)	Correa et al., 1997
			*r* = −0.76 (*P* < 0.05) (^1^56-day non-return)	Gillan et al., 2008
			*r* = −0.62 (*P* < 0.05) (^1^56-day non-return)	Morrell et al., 2018
IPIAH^8^	Percentage of IPIAH^8^ sperm	Fluorescence microscopy or flow cytometry	High IPIAH^8^ (44.5%): 64.7% of pregnancy rate^9^ Low IPIAH^8^ (8.5%): 36.2% of pregnancy rate^9^	Oliveira et al., 2014
ROS	Percentage of sperm cells producing ROS	Fluorescence microscopy or flow cytometry	High ROS (10.63%): low fertility rates Low ROS (6.11%): high fertility rates	Celeghini et al., 2019
DNA fragmentation	Percentage of sperm cells with DNA fragmentation	Fluorescence microscopy or flow cytometry	*r* = −0.56 (*P* < 0.01)	Morrell et al., 2018
Capacitation status	Percentage of acrosome-reacted spermatozoa	Fluorescence microscopy or flow cytometry	*r* = 0.37 (*P* < 0.05) (^1^litter size)	Kwon et al., 2015

*^1^Fertility measurement; ^2^wobble (%); ^3^progressive velocity (μm/s); ^4^lateral head amplitude (μm); ^5^beat cross frequency (Hz); ^6^linearity (%); ^7^path velocity (μm/s); ^8^spermatozoa with intact plasma and acrosome membranes and high mitochondrial membrane potential; ^9^ultrassonography exam 30 days after insemination.*

Several other techniques are currently available to assess sperm morpho-functional attributes. **Sperm motility, kinetics, vigor and hyperactivation levels**, are morpho-functional features classically assessed by light microscopy or computer-assisted sperm analysis (i.e., CASA) (Vincent et al., 2012). In addition, since sperm motility requires a substantial amount of energy produced by **mitochondrial activity** (Piomboni et al., 2012), high fertility samples usually present with high mitochondrial membrane potential. This feature can be assessed by fluorescence microscopy and flow cytometric approaches mainly using the JC-1 fluorescent probe, which forms J-aggregates when mitochondria are active and polarized; in this situation, more JC-1 monomers enter the spermatozoa and aggregate to generate red fluorescence (Gillan et al., 2005; Celeghini et al., 2007). In addition to energy production, mitochondria also produce large quantities of ROS that promote oxidative stress and damage to sperm membranes and DNA. Therefore, **ROS quantification** in sperm cells is also an important morpho-functional feature that can be assessed by fluorescence microscopy, flow cytometry and molecular/biochemical assays using fluorescent probes. Such assays include BODIPY, which measures the susceptibility of sperm to lipid peroxidation, CellROX Deep Red^®^, which stains ROS present in sperm cells, and measurement of malondialdehyde concentration (produced during lipid peroxidation) using the TBARS (Thiobarbituric acid reactive substances) assay (Aitken et al., 2007; Celeghini et al., 2019). In addition, since damage to the sperm membranes and DNA impairs sperm capacitation and early embryo development, both **plasma and acrosome membrane as well as DNA integrity** should be evaluated by fluorescence microscopy or flow cytometric approaches. Propidium iodide fluorescent probes and fluorescent lectin from *Pisum sativum* can be used to assess plasma and acrosome membrane damage (Celeghini et al., 2007), respectively, as well as SCSA^®^ (Sperm Chromatin Structure Assay) to detect the susceptibility of sperm to DNA fragmentation (Evenson and Wixon, 2006). Post-sperm **capacitation status** could be assessed using the combination of Hoechst 33258/chlortetracycline fluorescence probes and is proposed to predict litter size and fertility success in pigs (Kwon et al., 2015).

While the assessment of these features is essential to determine male fertility potential, based on data from human clinics, about 30% of sperm samples presenting with satisfactory morpho-functional features (Figure 3) are still unable to fertilize the oocytes or to trigger early embryo development. Recent advances in analyses of sperm molecular and subcellular content suggested that intrinsic factors may participate to early embryo development and, consequently, could be potential targets for the assessment of male fertility potential.

### Intrinsic Sperm Features

**FIGURE 4:**
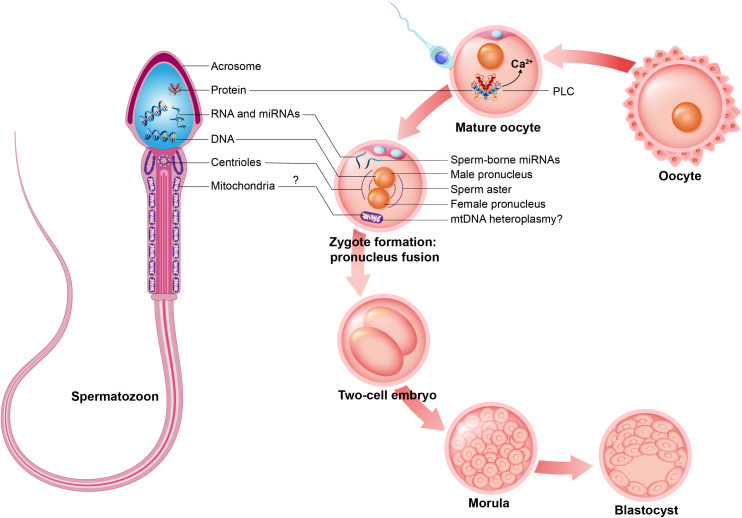
Intrinsic sperm features. Spermatozoon contributes early embryo development during mature oocyte fertilization, zygote formation and embryo cleavage potentially with intrinsic sperm features such as: sperm proteins (e.g., PLCζ, to promote oocyte activation); sperm RNAs and microRNAs (miRNAs); sperm DNA, to generate male pronucleus; sperm centrioles, to form sperm aster; and may contribute sperm mitochondria, promoting mtDNA heteroplasmy.

In addition to the paternal DNA, the spermatozoon carries numerous molecules and organelles, which constitute a growing focus of interest in fundamental research. Among these intrinsic factors, **sperm centrioles, sperm mitochondria, sperm proteins,** and **sperm RNAs** are particularly interesting with respect to their potential contribution to the post-fertilization process (Figure 4). As shown in Figure 2, sperm **centrioles** are localized in the sperm neck while sperm **mitochondria** are arranged in the midpiece. Since mature spermatozoa possess a minimal amount of cytoplasm and a nucleus with highly condensed DNA that prevents transcription, the presence of **proteins** and **RNAs** in sperm cells is limited. However, several proteins and ribonucleic acid molecules are likely acquired by the maturing spermatozoa during spermatogenesis and their transit through the epididymis and post-ejaculatory journey (Boerke et al., 2007).

## RNAs Species Are Major Sperm Intrinsic Factors

### microRNAs and Other Sperm RNA Species

Sperm RNAs were first described in the 1970s in murine and bovine spermatozoa (Betlach and Erickson, 1973; Paul and Duerksen, 1975). Given the limited quantity of cytoplasm and high level of sperm DNA condensation, this finding has been questioned as RNAs were potentially providing from mitochondria or contamination from somatic cells (Krawetz, 2005). In that regards, the roles and the origin of sperm RNAs remain to be determined (Ostermeier et al., 2004; Krawetz, 2005).

Four different RNA species can be distinguished in spermatozoa (Boerke et al., 2007): (1) mRNAs that are remnants from the spermatogenesis process with no known function in the oocyte (e.g., protamine-2 encoding RNA, Ziyyat, 2001); (2) mRNAs that originate from spermatogenesis with a potential function in the oocytes (e.g., PLCζ encoding RNA, Saunders et al., 2002); (3) mRNAs classified as “foreign” RNAs as a result of their acquisition by sperm during passage through the epididymis and by the contact with the fluids coming from the accessory sexual glands (e.g., clusterin encoding RNA, Hermo et al., 1994); and (4) non-coding RNAs (ncRNAs) that are acquired during the late steps of spermatogenesis or during post-testicular sperm maturation with a potential function at the time of fertilization (e.g., miRNAs and tRNA derived fragments, Sharma et al., 2016; Yuan et al., 2016). Among RNA species present in spermatozoa, ribosomal RNAs and mitochondrial RNAs (mtRNAs) are the most abundant, followed by ncRNAs. In contrast to somatic cells that comprise approximately 10 pg of RNA, mature spermatozoa carry around a 1,000-fold lower RNA content (Odia et al., 2018).

In addition to mRNAs, other sperm-borne RNA species, including miRNAs have been associated with sperm cell fertility status and early embryo development (Liu et al., 2012). The central dogma of molecular biology supports that genes are transcribed in the form of mRNAs, which in turn are translated into proteins, with the exception of structural RNAs such as ribosomal RNA (Mattick, 2001). Advancements in molecular biology and studies on the human genome have revealed that only 2% of all genes actually encode proteins (Mattick, 2001). In addition, many genomic sequences that were first described as “junk DNA” due to the fact that they did not encode proteins and had no apparent function, now appear to play an important role as post-transcriptional modulators. In this context, a large group of RNAs called non-coding RNAs (ncRNAs) has been identified in spermatozoa, including miRNAs (Wightman et al., 1993).

MicroRNAs are ∼18–22-nucleotide sequences that modulate gene expression following their pairing with the untranslated region (3′UTR) of target transcripts (Ambros, 2004; Bartel, 2004). The biosynthesis of miRNAs is performed in the cell nucleus with the generation of primary miRNA transcripts (pri-miRNA) and then precursor miRNAs (pre-miRNA), which are transported to the cytoplasm. In the cytoplasm, the hairpin structure is cleaved by Dicer, resulting in small double-stranded transcripts. The miRNA double strand is then transferred to Argonaut proteins, which bind to the mature miRNA also called guide-miRNA. This complex promotes gene silencing through translational inhibition or cleavage of mRNA (Ambros, 2004; Bartel, 2004). Such cleavage is common in plants and occurs when the pairing between the miRNA and 3′UTR region is perfect. On the other hand, an imperfect pairing implies the blockade of translation machinery on that particular mRNA without degradation—the most common process in animals (Bartel, 2004, 2018).

Of interest is that miRNAs either act in the cell where they are transcribed or in target cells through an intercellular communication mechanism (Raposo and Stoorvogel, 2013). Extracellular vesicles are important mediators of this communication. Exosomes comprise a population of small (40–160 nm) extracellular vesicles derived from intracellular multivesicular bodies carrying diverse bioactive molecules, including miRNAs, mRNAs and proteins (Raposo and Stoorvogel, 2013). Once exosomes are released into the extracellular environment, they can be internalized by recipient target cells (Raposo and Stoorvogel, 2013). Exosomes are released from different internal organs of the male reproductive tract, including the epididymis (epididymosomes) and prostate (prostasomes) (Sullivan and Saez, 2013). Epididymosomes play a key role during cellular communication that promotes sperm maturation during spermatozoa passage into the microenvironment of the epididymis (Belleannée, 2015).

**FIGURE 5:**
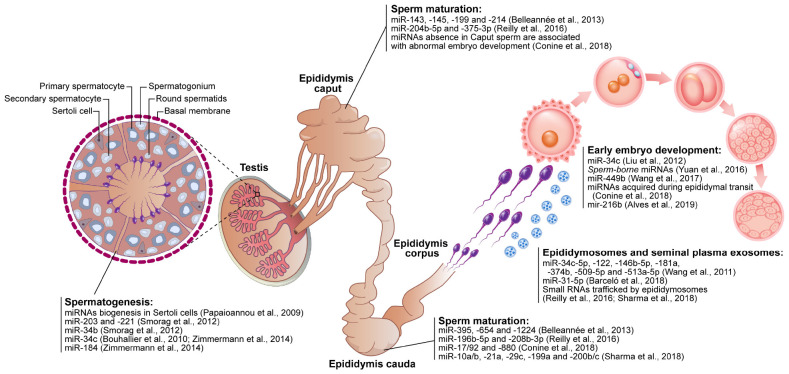
Schematic figure demonstrating the contribution of microRNAs (miRNAs) in the reproductive events. The sperm-related-miRNAs molecules display functions in spermatogenesis (testis), sperm maturation (epididymis *caput*, *corpus*, and *cauda*), sperm and seminal plasma interaction (e.g., epididymosomes) as well as modulating early embryo development.

In general, miRNAs are post-transcriptional regulators that play important roles in physiological processes and their impairment results in different pathologies. These molecules have been shown to have great importance in the regulation of spermatogenesis (Papaioannou et al., 2009; Kotaja, 2014) and sperm maturation during sperm passage through the epididymis (Björkgren et al., 2012; Jerczynski et al., 2016; Sipilä and Björkgren, 2016) as shown in Figure 5. With the knowledge that miRNAs are very stable, conserved bioactive molecules derived from the different internal organs of the male reproductive tract, they could be considered potent fertility biomarkers. In light of these findings, sperm cells have gained the status of gametes that are not only responsible for transporting genetic material, but also as contributors to the activation of the oocyte and the cellular structure and molecular components of the zygote as shown in Figure 5.

### miRNAs and Spermatogenesis

Spermatogenesis is a well-organized process that culminates in the production of sperm cells. Conditional knock-out of the miRNA processing enzyme Dicer1 in Sertoli cells triggers male infertility due to the absence of sperm in the lumen of the seminiferous tubules and spermatogenesis disorders (Papaioannou et al., 2009; Zimmermann et al., 2014), suggesting that the biogenesis of small non-coding RNA is important to spermatogenesis. Indeed, each cell type of the seminiferous tubule has a different miRNA profile that plays an important role in proliferation and differentiation (Hayashi et al., 2008; Smorag et al., 2012). In pigs, miR-26a has been shown to inhibit proliferation and to promote apoptosis in Sertoli cells, thus impairing sperm production (Ran et al., 2018). In mice, miR−221 and miR−290 regulate the proliferation of spermatogonia and primary spermatocytes (Smorag et al., 2012). In addition, miR−203 modulates spermatocyte meiosis and the miR−34 family regulates the formation of spermatids (Smorag et al., 2012). In humans, pigs and mice, the miR−34 family is also important in the regulation of spermatogenesis (Bouhallier et al., 2010; Zimmermann et al., 2014). Thus, miRNAs such as miR−26a, miR−221, miR−290, miR−203, and the miR−34 have been proposed to contribute to spermatozoa production (Figure 5), as their dysregulation results in disturbances in spermatogenesis and consequently a decrease in male fertility potential.

### miRNAs and Sperm Maturation

Once produced in the testis, spermatozoa must pass through the epididymis to acquire their motility and oocyte-fertilizing ability. During their passage through the different epididymal segments, spermatozoa come into contact with different repertoires of ncRNAs, transcripts and proteins that are released from the epithelium of the epididymis mostly via epididymosomes (Sullivan et al., 2007; Belleannée et al., 2013; Sullivan, 2015; Reilly et al., 2016; Sharma et al., 2018). The transfer of proteins and other contents from epididymosomes to spermatozoa has been proposed to occur via the dynamin 1 mechanoenzyme following the tethering of epididymosomes to specific sperm receptors located in the post-acrosomal region and the fusion of epididymosomes with the sperm membrane (Zhou et al., 2019). In mice, sperm miRNAs present in the sperm are altered along the epididymis (Figure 5), showing that the sperm miRNA content is dynamic even following spermatogenesis (Nixon et al., 2015).

Conditional knock-out mice for the *Dicer* enzyme in principal cells of the epididymis dysregulated the differentiation process (Björkgren et al., 2012). Thus, biogenesis of ncRNAs seems to be important in the regulation of epididymal epithelium, sperm maturation and fertility. While miR-10a/b, −21a, −29c, −196b−5p, −199a, −200b/c, and −208b−3p accumulate in spermatozoa during passage through the epididymis, miR−204b−5p and miR−375−3p are more abundant in spermatozoa from the *caput* and *corpus* of the epididymis (Reilly et al., 2016; Sharma et al., 2018). Epididymosomes also display different miRNA profiles and traffic small RNAs to spermatozoa (Sharma et al., 2018), including miR−143, −145, −199, and −214 that are more abundant in epididymosomes present in the *caput* of the bovine epididymis, and miR−395, −654, and −1224 that are more abundant in epididymosomes from the *cauda* (Belleannée et al., 2013). In men with reproductive abnormalities, miRNAs present in seminal plasma (miR−34c−5p, −122, −146b−5p, −181a, −374b, −509−5p, and −513a−5p) decreased in abundance in men presenting with azoospermia (absence of spermatozoa in the ejaculate) and increased in abundance in men presenting with asthenozoospermia (decreased sperm motility) (Wang et al., 2011). Although studies have shown the importance of miRNAs along the epididymis, further work is required to show how miRNAs are able to regulate sperm maturation during passage of spermatozoa through the epididymis and how regulation of motility acquisition and the ability of the sperm to become fertile occur.

## Contribution of Sperm Intrinsic Factors to Post-Fertilization Processes

**FIGURE 6:**
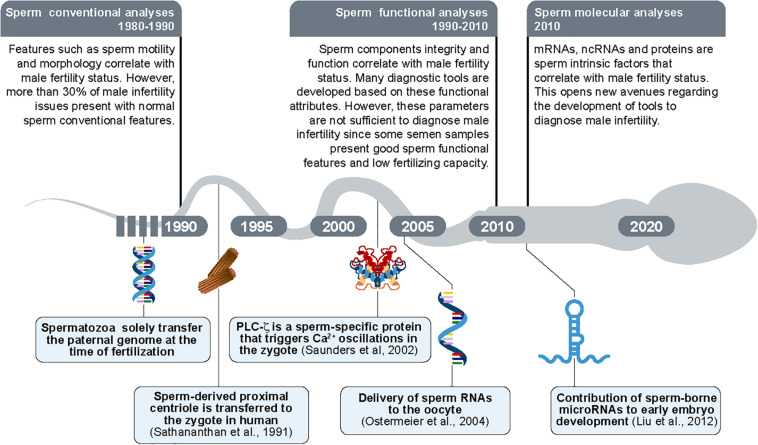
Timeline of the new findings regarding sperm morpho-functional and intrinsic features. The paternal DNA was considered as the sole intrinsic sperm feature transferred from spermatozoon to oocyte until 1990s. In parallel, the sperm evaluation was limited to sperm conventional analyses (sperm motility and sperm morphology/abnormalities). Proximal centrioles were then shown to be transmitted by sperm to the oocytes during fertilization for the first time in 1991 (Sathananthan et al., 1991). The sperm-borne PLCζ protein was shown as a promotor of oocyte activation in 2002 (Saunders et al., 2002). The delivery of RNAs molecules was then revealed as transferred from sperm to the oocyte in 2004 (Ostermeier et al., 2004). In parallel, the sperm evaluation was updated to sperm functional analyses (e.g., sperm plasma membrane integrity). In 2010s, small sperm-borne RNAs molecules (microRNAs/miRNAs) were shown as important to early embryo development (Liu et al., 2012) potentially constituting a new group of sperm analyses composed by evaluation of molecular targets.

For many years it was postulated that sperm cells had the exclusive function of transporting genetic material to the oocyte. However, at the end of the 20th century, this sole contribution was challenged by the fact that specific sperm intrinsic factors, including sperm centrioles, sperm proteins, ribonucleic acids and mitochondrial DNA (mtDNA) could contribute to fertilization and/or early embryo development (Figure 6).

### Contribution of the Sperm Centriole to Embryo Development

Centrioles are cytoplasmic structures involved in cell division and in the formation of cilia and flagella (Avidor-Reiss and Fishman, 2019). While spermatozoa present with centrioles arranged in the neck of their tail (Figure 2), mature oocytes lack centrioles (Simerly et al., 2018). Therefore, sperm-derived centrioles ensure the formation of sperm aster and centrosome in the fertilized oocyte, which bring the female and male pronuclei into close proximity and promote syngamy (Simerly et al., 1995; Schatten and Sun, 2009), as shown in Figure 4. Thus, centrioles are organelles that ensure proper embryonic development. In addition, the proper formation of the sperm aster is an important factor related to male fertility. For instance, large and well organized sperm aster formations in human spermatozoa correlate with samples that present with high fertility, while small and disorganized sperm aster formations correlate with low fertility samples (Navara et al., 1997). Therefore, beyond the transmission of the centrioles by the sperm cells, the quality of the sperm-borne centrioles is also important to embryo development. It has recently been shown that sperm from non-rodent mammals are responsible for transmitting a second atypical centriole to oocyte (Fishman et al., 2018). This centriole potentially forms the sperm aster and centrosome in the oocytes and, together with the first centriole, supports the fusion between the female and male pronucleus.

### The Questioned Contribution of Sperm Mitochondrial DNA to Embryo Paternal Inheritance

Mitochondria are peculiar organelles that possess a specific DNA type: mtDNA. This organelle is essentially responsible for energy production, which is important to the cell’s activity. During spermatogenesis, sperm mitochondria change from a conventional to a condensed form that ensures more efficient energy production (Amaral et al., 2013). While some mitochondria are lost during spermatogenesis, between 22 and 75 mitochondria remain in the midpiece of the mature spermatozoon depending on species (Figure 2) (Bahr and Engler, 1970; Song and Lewis, 2008). Although spermatic mitochondria also contain mtDNA, the current paradigm is that the embryo exclusively possesses maternal mitochondria and, consequently, mitochondrial diseases are only inherited from the mother (Hecht et al., 1984). However, the inheritance of paternal mtDNA was suggested following the discovery of a high level of mtDNA heteroplasmy, i.e., mix of maternal and paternal mitochondrial genome, in mouse (Gyllensten et al., 1991) and human (17 individuals) (Luo et al., 2018) as schematized in Figure 4. These findings open new research avenues to better understand mitochondrial disease inheritance. However, further studies are necessary to confirm this potential paradigm shift.

### Contribution of Sperm Proteins to Oocyte Activation

While mature spermatozoa are transcriptionally inactive upon their entrance to the epididymis, their proteome is dynamic due to the acquisition of proteins from the surrounding fluid, the cleavage of others from the sperm surface, and the rearrangement of acrosomal proteins during sperm capacitation. These proteins ensure diverse functions at different stages including: **sperm maturation**, e.g., the glioma pathogenesis-related 1-like protein 1 (GLiPr1L1) that is acquired by spermatozoa and participates in the binding of sperm cells to zona pellucida (Caballero et al., 2012); **tagging of sperm sub-populations to ensure heterogeneity**, e.g., the sperm binding protein 1 (ELSPBP1) that discriminates dead spermatozoa (D’Amours et al., 2012); and **sperm ability to bind to the oocyte**, e.g., Spermadhesin AWN (AWN) that promotes sperm binding to oocyte’s zona pellucida during fertilization (Kwon et al., 2014).

In addition to these proteins, sperm cells also acquire proteins during the final steps of spermatogenesis that are important to **oocyte activation**, e.g., the sperm-specific WW domain-binding protein (PAWP) and phospholipase C zeta (PLCζ) (Saunders et al., 2002; Wu et al., 2007). Among these spermatic proteins, PLCζ is an important player during fertilization, since it triggers intracellular calcium impulses required for oocyte activation (Saunders et al., 2002; Kashir et al., 2010). As a result of these impulses, and once fertilized, the metaphase II mature oocyte is able to resume and progress through meiotic division. This ensures the formation of female and male pronuclei, followed by syngamy (Figure 4). The presence of these proteins in spermatozoa positively correlates with male fertility (Saunders et al., 2002; Aarabi et al., 2014).

### Contribution of Sperm RNA Species to Embryo Development

#### Sperm RNAs

The detection of sperm-borne RNAs, including *clusterin* and *AKAP4*, in embryos raised questions regarding their potential contribution to the molecular regulation of embryonic development (Ostermeier et al., 2004). The discovery of zygote transcripts originating exclusively from spermatozoa opened new avenues into the investigation of sperm RNAs and their importance to embryo development (Ostermeier et al., 2005; Jodar et al., 2013). Most sperm RNAs are transcribed and then stored or inactivated in spermatozoa during spermiogenesis. During this stage of spermatogenesis, histones are replaced by protamines in sperm DNA, changing the shape of nucleosomes to a toroid structure with high sperm nuclear condensation culminating in gene expression silencing. However, 1% of histones in mice and 10–15% of histones in humans and cattle remain in the form of nucleosomes in spermatozoa (Samans et al., 2014). The remaining nucleosomes in human and bovine spermatozoa are located in similar promoter regions, indicating transcriptional conservation of specific genes related to early embryonic development (Samans et al., 2014). Even if the presence of RNAs is now accepted in spermatozoa, there is no evidence showing that RNAs can be transcribed from DNA in mature spermatozoa. Most of the RNAs present in sperm cells are thus potentially acquired during spermatogenesis as well as during passage through the epididymis.

Even though sperm cells possess limited quantities of sperm RNAs, sperm-borne RNAs have been associated with many relevant reproductive processes and several correlate with fertility potential in the bull, including AK1 (adenylate kinase 1; *R*^2^ = 0.90), IB (integrin β5, *R*^2^ = 0.95), NGF (*R*^2^ = 0.47), TIMP (tissue inhibitor of metalloproteinases, *R*^2^ = 0.39), SNRPN (small nuclear ribonucleoprotein polypeptide N, *R*^2^ = 0.71) and PLCζ (phospholipase C, *R*^2^ = 0.69) (Kasimanickam et al., 2012). Similarly, the presence of 415 transcripts out of 24,000, were found in different proportions between bovine spermatozoa presenting with high or low fertility potential (Feugang et al., 2010). In human sperm cells, ANXA2 (Annexin A2), BRD2 (Bromodomain Containing 2), OAZ3 (Ornithine Decarboxylase Antizyme 3), PRM1 (Protamine 1) and PRM2 (Protamine 2) transcripts were present in lower amounts in sperm cells with low motility (Jodar et al., 2013).

#### Sperm miRNAs

Until embryonic gene activation, i.e., when the embryo begins to transcribe its newly formed genome, embryo development relies on the expression of maternal transcripts originating from the oocyte and, to a lesser extent, from spermatozoa. MicroRNAs are also delivered by gametes to the embryos. Until activation of the embryonic genome the abundance of miRNA transcripts present in the embryo decreases until embryonic gene activation begins (Tang et al., 2007). Murine embryos produced from *Dicer* enzyme knock-out oocytes fail to undergo the first cell division, demonstrating the importance of the miRNAs or other ncRNAs contributed by the female gamete during early embryonic development (Tang et al., 2007). Similarly, murine embryos generated with normal oocytes and *Dicer* or *Drosha* enzyme knock-out spermatozoa showed decreased development rates, which recovered following the replacement of miRNAs (Yuan et al., 2016). In addition, the inhibition of miR-34c, which is exclusively delivered by sperm to the embryo in mice (Liu et al., 2012), but not in cattle (Tscherner et al., 2014), prevents the first cleavage (Liu et al., 2012). Sperm-borne miR−449b overexpression in cloned embryos is associated with enhanced cleavage rates at the 8-cell stage and lower rates of apoptosis in the blastocyst (Wang et al., 2017). Sperm-borne miR−216b was recently shown to be present at higher abundance in spermatozoa from low fertility bulls and is associated with a reduced first cleavage rate as well as a reduced number of cells in blastocysts (Alves et al., 2019). In parallel, the RNAs that are acquired by spermatozoa during epididymal transit were shown to be essential for embryo implantation and development in mice (Conine et al., 2018, 2019). Thus, despite the decrease in miRNA levels until activation of the embryonic genome, proper embryo development potentially relies on miRNAs delivered by the gametes (Figure 5).

### Contribution of Small Non-coding RNAs to Transgenerational Inheritance of Traits

The transgenerational inheritance of traits concerns the transmission of characteristics from the parents to the offspring, i.e., heritable phenotype, without altering the DNA sequence (Weinhold, 2006). Following advances in the field of epigenetics, i.e., the study of inheritance that is not based on DNA sequence, but on how the DNA sequence is utilized, miRNAs transmitted by spermatozoa at the time of fertilization are considered epigenetic factors that have some effects on the offspring.

Environmental conditions such as toxicant exposure (Schuster et al., 2016), mental stress (Rodgers et al., 2013), alcohol consumption (Rompala et al., 2018), and low protein or high fat diet (Sharma et al., 2016) are considered principal modulators of epigenetic markers by promoting alterations in the sperm “epigenome.” Rodgers et al. (2015) identified nine miRNAs that had altered abundance in the sperm cells from mice submitted to chronic stress. Surprisingly, the injection of these nine miRNAs into embryos of non-stressed parents recapitulated the effects observed in parents undergoing chronic stress (Rodgers et al., 2015). In a similar manner, regulation of another group of ncRNAs in sperm cells, the tRNA-derived fragments, was dependent on the diet of the sire and this influenced the offspring phenotype (Sharma et al., 2016). These studies show that sperm-borne ncRNAs potentially own epigenetic influence. However, further studies are necessary in order to explain the mechanism of action of these molecules in the offspring.

## Linking miRNAs to Male (In)Fertility Status

Since miRNAs play an important role in the physiological processes related to spermatogenesis, sperm maturation and early embryonic development (Figure 5), these molecules have the potential to modulate sperm morpho-functional features as well as male fertility ability. In that regards, different miRNA profiles have been associated with different sperm quality levels and fertility phenotypes from animal models and human clinical samples as listed in Table 2.

**TABLE 2:** Sperm miRNAs associated with different infertility issues in porcine, human, bovine and mouse.

**miRNAs**	**Abundance**	**Phenotype associate**	**Specie**	**References**
let-7a	Down-regulated	Low abnormalities	Porcine	Curry et al., 2011
let-7a-5p	Down-regulated	High fertility	Human	Salas-Huetos et al., 2016
let-7d	Down-regulated	Low abnormalities/High motility	Porcine	Curry et al., 2011
let-7e	Down-regulated	Low abnormalities/High motility	Porcine	Curry et al., 2011
let-7f-5p	Down-regulated	High fertility	Human	Salas-Huetos et al., 2016
miR-9-3p	Down-regulated	High fertility	Human	Salas-Huetos et al., 2016
miR-15a	Down-regulated	High fertility	Bovine	Menezes et al., 2019
miR-15b	Up-regulated	Low abnormalities	Porcine	Curry et al., 2011
miR-15b	Down-regulated	Oligoasthenozoospermic	Human	Abu-Halima et al., 2013
miR-16	Down-regulated	Oligoasthenozoospermic	Human	Abu-Halima et al., 2013
miR-17-5p	Up-regulated	High motility sperm	Bovine	Capra et al., 2017
miR-19a	Down-regulated	Oligoasthenozoospermic	Human	Abu-Halima et al., 2013
miR-19b-3p	Down-regulated	High fertility	Bovine	Fagerlind et al., 2015
miR-20a-5p	Up-regulated	High motility sperm	Bovine	Capra et al., 2017
miR-22	Down-regulated	Low abnormalities	Porcine	Curry et al., 2011
miR-22-3p	Down-regulated	High fertility	Human	Salas-Huetos et al., 2016
miR-26a-5p	Up-regulated	High motility sperm	Bovine	Capra et al., 2017
miR-27a-5p	Down-regulated	High fertility	Bovine	Fagerlind et al., 2015
miR-29b	Down-regulated	High fertility	Bovine	Menezes et al., 2019
miR-30b-5p	Down-regulated	High fertility	Human	Salas-Huetos et al., 2016
miR-30d-3p	Down-regulated	High fertility	Human	Salas-Huetos et al., 2016
miR-33b	Up-regulated	High fertility	Bovine	Alves et al., 2019
miR-34a-5p	Up-regulated	High fertility	Human	Salas-Huetos et al., 2016
miR-34b	Down-regulated	Oligoasthenozoospermic	Human	Abu-Halima et al., 2013
miR-34c	Up-regulated	High fertility	Mouse	Liu et al., 2012
miR-34c-5p	Down-regulated	Oligoasthenozoospermic	Human	Abu-Halima et al., 2013
miR-34c-3p	Down-regulated	High fertility	Bovine	Fagerlind et al., 2015
miR-93-3p	Down-regulated	High fertility	Human	Salas-Huetos et al., 2016
miR-103a-3p	Down-regulated	High fertility	Human	Salas-Huetos et al., 2016
miR-122	Down-regulated	Oligoasthenozoospermic	Human	Abu-Halima et al., 2013
miR-122-5p	Down-regulated	High motility sperm	Bovine	Capra et al., 2017
miR-126-5p	Up-regulated	High fertility	Bovine	Alves et al., 2019
miR-127	Down-regulated	Capacitated sperm	Porcine	Li et al., 2018
miR-130b-5p	Down-regulated	High fertility	Human	Salas-Huetos et al., 2016
miR-132-5p	Down-regulated	High fertility	Human	Salas-Huetos et al., 2016
miR-145-5p	Down-regulated	High fertility	Human	Salas-Huetos et al., 2016
miR-148b-3p	Down-regulated	High fertility	Bovine	Fagerlind et al., 2015
miR-149-3p	Down-regulated	High fertility	Human	Salas-Huetos et al., 2016
miR-151-3p	Up-regulated	Capacitated sperm	Porcine	Li et al., 2018
miR-152	Down-regulated	Capacitated sperm	Porcine	Li et al., 2018
miR-181a-3p	Down-regulated	High fertility	Human	Salas-Huetos et al., 2016
miR-184	Down-regulated	High motility sperm	Bovine	Capra et al., 2017
miR-193b-5p	Down-regulated	High fertility	Human	Salas-Huetos et al., 2016
miR-205	Up-regulated	High fertility	Bovine	Alves et al., 2019
miR-208a	Up-regulated	High fertility	Human	Salas-Huetos et al., 2016
miR-212-3p	Up-regulated	High fertility	Human	Salas-Huetos et al., 2016
miR-216b	Down-regulated	High fertility	Bovine	Alves et al., 2019
miR-222-5p	Down-regulated	High fertility	Human	Salas-Huetos et al., 2016
miR-296-5p	Down-regulated	High fertility	Human	Salas-Huetos et al., 2016
miR-320a	Down-regulated	High fertility	Bovine	Fagerlind et al., 2015
miR-324-3p	Up-regulated	High fertility	Human	Salas-Huetos et al., 2016
miR-339a	Down-regulated	High fertility	Bovine	Alves et al., 2019
miR-339-5p	Down-regulated	High fertility	Human	Salas-Huetos et al., 2016
miR-340-3p	Down-regulated	High fertility	Human	Salas-Huetos et al., 2016
miR-346	Down-regulated	High fertility	Human	Salas-Huetos et al., 2016
miR-365a-3p	Down-regulated	High fertility	Human	Salas-Huetos et al., 2016
miR-432-3p	Down-regulated	High fertility	Human	Salas-Huetos et al., 2016
miR-449a	Down-regulated	Oligoasthenozoospermic	Human	Abu-Halima et al., 2013
miR-483-5p	Up-regulated	High fertility	Human	Salas-Huetos et al., 2016
miR-486-5p	Down-regulated	High motility sperm	Bovine	Capra et al., 2017
miR-487a	Down-regulated	High fertility	Human	Salas-Huetos et al., 2016
miR-491-5p	Up-regulated	High fertility	Human	Salas-Huetos et al., 2016
miR-500	Up-regulated	High fertility	Bovine	Alves et al., 2019
miR-502-5p	Down-regulated	High fertility	Bovine	Fagerlind et al., 2015
miR-505	Up-regulated	High fertility	Bovine	Alves et al., 2019
miR-517-5p	Down-regulated	High fertility	Human	Salas-Huetos et al., 2016
miR-518d-5p	Down-regulated	High fertility	Human	Salas-Huetos et al., 2016
miR-518f-3p	Up-regulated	High fertility	Human	Salas-Huetos et al., 2016
miR-520c-3p	Down-regulated	High fertility	Human	Salas-Huetos et al., 2016
miR-520d-3p	Up-regulated	High fertility	Human	Salas-Huetos et al., 2016
miR-526b-5p	Down-regulated	High fertility	Human	Salas-Huetos et al., 2016
miR-532	Up-regulated	High fertility	Bovine	Alves et al., 2019
miR-542-5p	Up-regulated	High fertility	Bovine	Alves et al., 2019
miR-543	Down-regulated	High fertility	Human	Salas-Huetos et al., 2016
miR-552	Down-regulated	High fertility	Human	Salas-Huetos et al., 2016
miR-564	Up-regulated	High fertility	Human	Salas-Huetos et al., 2016
miR-573	Down-regulated	High fertility	Human	Salas-Huetos et al., 2016
miR-596	Down-regulated	High fertility	Human	Salas-Huetos et al., 2016
miR-622	Down-regulated	High fertility	Human	Salas-Huetos et al., 2016
miR-636	Up-regulated	High fertility	Human	Salas-Huetos et al., 2016
miR-644a	Down-regulated	High fertility	Human	Salas-Huetos et al., 2016
miR-659-3p	Down-regulated	High fertility	Human	Salas-Huetos et al., 2016
miR-708-5p	Up-regulated	High fertility	Human	Salas-Huetos et al., 2016
miR-744-5p	Down-regulated	High fertility	Human	Salas-Huetos et al., 2016
miR-766-3p	Down-regulated	High fertility	Human	Salas-Huetos et al., 2016
miR-935	Down-regulated	High fertility	Human	Salas-Huetos et al., 2016
miR-942	Down-regulated	High fertility	Human	Salas-Huetos et al., 2016
miR-1249	Down-regulated	High fertility	Bovine	Fagerlind et al., 2015
miR-1254	Up-regulated	High fertility	Human	Salas-Huetos et al., 2016
miR-1285	Down-regulated	Capacitated sperm	Porcine	Li et al., 2018
miR-1296	Down-regulated	High fertility	Human	Salas-Huetos et al., 2016
miR-1298	Down-regulated	High fertility	Human	Salas-Huetos et al., 2016
miR-1973	Up-regulated	Asthenozoospermic	Human	Abu-Halima et al., 2013
miR-1973	Down-regulated	Oligoasthenozoospermic	Human	Abu-Halima et al., 2013

Functional studies have been carried out in knock-out mouse models to determine the contribution of individual miRNAs to male fertility (Comazzetto et al., 2014; Wu et al., 2014). For instance, simultaneous deletion of miR34b/c and miR−449 triggered male infertility following the impairment of spermatogenesis, motile ciliogenesis, and sperm maturation (Comazzetto et al., 2014; Wu et al., 2014). While targeted deletion is essential to unravel the function of specific miRNAs *in vivo*, phenotypic effects are rarely observed after invalidation of a single miRNA-encoding gene due to compensatory functions of related miRNAs. These functional approaches were complemented by several correlative studies performed in large mammals and humans.

Within livestock animals, bulls that presented satisfactory sperm quality and history of distinct fertility (high *vs.* low fertility) displayed different profiles of sperm miRNAs that are summarized in Table 2 (Fagerlind et al., 2015; Alves et al., 2019; Menezes et al., 2019). Similarly, spermatozoa from boar featuring low motility and a high percentage of abnormalities, displayed a higher abundance of the miRNAs: let−7a, let−7d, let−7e, and miR−22; and lower abundance of miR−15b (Curry et al., 2011) compared with spermatozoa presenting with normal features. In addition, Capra et al. (2017) sorted spermatozoa according to their motility and showed that miR−17−5p, −20a−5p, −26a−5p, −122−5p, −184, and −486−5p were differently displayed between the two subpopulations (Capra et al., 2017). Capacitated and non-capacitated spermatozoa also appeared to show different miRNAs profiles. For instance, Li et al. (2018) showed that miR−127, −151−3p, −152, −1285 were differently expressed in *in natura* (fresh) compared to capacitated boar sperm (Li et al., 2018). Based on that sperm miRNAs features, variation in expression of miRNAs associated with economically important infertility issues might open avenues to the development of selection programs in livestock animals. Targeting seminal miRNAs candidates as non-invasive biomarkers for male infertility could thus have profound economic impact on livestock industry.

In humans, sperm miRNA profiles from fertile and infertile men accompanied or not by morpho-functional sperm alterations have been extensively investigated. In that regards, men with reproductive disorders presented different sperm miRNA profiles compared with normospermic control individuals: *asthenozoospermia* (low sperm motility) was associated with higher abundance of 50 sperm miRNAs, among then hsa-miR−34b, −122, and −1973, and lower abundance of 27 sperm miRNAs; *oligoasthenozoospermia* (low sperm concentration and low sperm motility) was associated with higher abundance of 42 miRNAs (hsa-miR−15b, −16, −19a, −34b, −34c−5p, −122, −449a e −1973 were the most abundant) and lower abundance of 44 miRNAs (Abu-Halima et al., 2013); *teratozoospermia* (abnormal sperm morphology) was associated with the downregulation of six miRNAs (hsa-miR−151−5p, −935, −125a−3p, −132−5p, −320b, −195−5p) (Salas-Huetos et al., 2015). On the other hand, the sperm miRNA profile of men who presented normal semen quality (normozoospermic), but had a different fertility performance (fertile *vs.* infertile) showed that 57 miRNAs were presented at a different abundance between the groups. Among these miRNAs miR-15b−5p and 34a−5p, were related to pathways linked to embryonic development and chromatin remodeling (Salas-Huetos et al., 2016). Correlations were also observed between sperm miRNA content and assisted reproductive technology (ART) outcomes. For instance, the study from Cui et al. (2015) indicated that the proportion of good quality embryos obtained after 3 days post-ICSI positively correlated with the amount of hsa-miR−34c found in spermatozoa (Cui et al., 2015). While several confounding factors might be considered before drawing extrapolative conclusions, some spermatic miRNAs may thus become predictive biomarker of defective spermatozoa and ART outcome. Tentative explanations have been proposed regarding the molecular mechanism involved in these correlative observations between sperm miRNA profiles and fertility levels. For instance, hsa-miR−27a that is increased in asthenoteratozoospermic patients regulates the post-transcriptional expression of the Cysteine-Rich Secretory Protein2 (CRISP2), which encodes for a protein playing a role in sperm motility, acrosome reaction and gamete fusion (Zhou et al., 2017).

Overall, the different studies performed on human clinical samples as well as on animal models have shown that different profiles of sperm miRNA content can be observed according to sperm morpho-functional features and fertility status. Extracellular miRNAs correlated with male infertility issues are also retrieved in seminal plasma (Barceló et al., 2018), which encompasses secretions from the different internal organs of the male reproductive tract. While both spermatic and extra-cellular RNA could be used as molecular targets for the non-invasive diagnosis of male infertility and reproductive system diseases, a better understanding of miRNA-mediated regulation of reproductive functions is needed.

## Final Considerations

In summary, sperm cells should display specific factors to fulfill the “healthy sperm” concept, including proper morpho-functional features in addition to intrinsic factors. While sperm cell morpho-functional attributes are not always sufficient to predict male fertility potential, spermatozoa carry different factors, including organelles (centrioles, mitochondria) and molecules (proteins, RNAs, ncRNAs), which are involved in important steps of reproductive biology, e.g., spermatogenesis, sperm maturation, fertilization and embryo development. These factors constitute potential biomarkers of “healthy sperm” and male fertility status and may become major assets for diagnosing instances of idiopathic male infertility in both humans and livestock animals. A better understanding of the mechanism of action of these sperm intrinsic factors in the regulation of reproductive and developmental processes still presents a major challenge that must be addressed.

## Author Contributions

CB, MA, and EC wrote the manuscript and designed the figures. All the authors revised the manuscript.

## Conflict of Interest

The authors declare that the research was conducted in the absence of any commercial or financial relationships that could be construed as a potential conflict of interest.

## References

[B1] Aarabi M. Balakier H. Bashar S. Moskovtsev S. I. Sutovsky P. Librach C. L. (2014). Sperm content of postacrosomal WW binding protein is related to fertilization outcomes in patients undergoing assisted reproductive technology. *Fertil. Steril.* 102 440–447. 10.1016/j.fertnstert.2014.05.003 24907910

[B2] Abu-Halima M. Hammadeh M. Schmitt J. Leidinger P. Keller A. Meese E. (2013). Altered microRNA expression profiles of human spermatozoa in patients with different spermatogenic impairments. *Fertil. Steril.* 99 1249.e16–1255.e16. 10.1016/j.fertnstert.2012.11.054 23312218

[B3] Agarwal A. Mulgund A. Hamada A. Chyatte M. R. (2015). A unique view on male infertility around the globe. *Reprod. Biol. Endocrinol.* 13 1–9. 10.1186/s12958-015-0032-1 25928197 PMC4424520

[B4] Aitken R. J. Wingate J. K. De Iuliis G. N. McLaughlin E. A. (2007). Analysis of lipid peroxidation in human spermatozoa using BODIPY C11. *MHR Basic Sci. Reprod. Med.* 13 203–211. 10.1093/molehr/gal119 17327268

[B5] Alves M. B. R. de Arruda R. P. De Bem T. H. C. Florez-Rodriguez S. A. Sá Filho M. F. de Belleannée C. (2019). Sperm-borne miR-216b modulates cell proliferation during early embryo development via K-RAS. *Sci. Rep.* 9 1–14. 10.1038/s41598-019-46775-8 31316130 PMC6637201

[B6] Amann R. P. Hammerstedt R. H. (1993). In vitro evaluation of sperm quality: an opinion. *J. Androl.* 14 397–406. 10.1002/j.1939-4640.1993.tb03247.x 8294222

[B7] Amaral A. Lourenço B. Marques M. Ramalho-Santos J. (2013). Mitochondria functionality and sperm quality. *Reproduction* 146 163–174. 10.1530/REP-13-0178 23901129

[B8] Ambros V. (2004). The functions of animal microRNAs. *Nature* 431 350–355. 10.1038/nature02871 15372042

[B9] Avidor-Reiss T. Fishman E. L. (2019). It takes two (centrioles) to tango. *Reproduction* 157 R33–R51. 10.1530/REP-18-0350 30496124 PMC6494718

[B10] Bahr G. F. Engler W. F. (1970). Considerations of volume, mass, DNA, and arrangement of mitochondria in the midpiece of bull spermatozoa. *Exp. Cell Res.* 60 338–340. 10.1016/0014-4827(70)90526-4 5422964

[B11] Barceló M. Mata A. Bassas L. Larriba S. (2018). Exosomal microRNAs in seminal plasma are markers of the origin of azoospermia and can predict the presence of sperm in testicular tissue. *Hum. Reprod*. 33 1087–1098. 10.1093/humrep/dey072 29635626 PMC5972609

[B12] Bartel D. P. (2004). MicroRNAs: genomics, biogenesis, mechanism, and function. *Cell* 116 281–297. 10.1016/S0092-8674(04)00045-5 14744438

[B13] Bartel D. P. (2018). Metazoan MicroRNAs. *Cell* 173 20–51. 10.1016/j.cell.2018.03.006 29570994 PMC6091663

[B14] Belleannée C. (2015). Extracellular microRNAs from the epididymis as potential mediators of cell-to-cell communication. *Asian J. Androl.* 17 730–736. 26178395 10.4103/1008-682X.155532 PMC4577581

[B15] Belleannée C. Calvo É Caballero J. Sullivan R. (2013). Epididymosomes convey different repertoires of microRNAs throughout the bovine epididymis. *Biol. Reprod.* 89:30. 10.1095/biolreprod.113.110486 23803555

[B16] Betlach C. Erickson R. (1973). A unique RNA species from maturing mouse spermatozoa. *Nature* 242 114–115. 10.1038/242114a0 4694296

[B17] Bianchi E. Doe B. Goulding D. Wright G. J. (2014). Juno is the egg Izumo receptor and is essential for mammalian fertilization. *Nature* 508 483–487. 10.1038/nature13203 24739963 PMC3998876

[B18] Björkgren I. Saastamoinen L. Krutskikh A. Huhtaniemi I. Poutanen M. Sipilä P. (2012). Dicer1 ablation in the mouse epididymis causes dedifferentiation of the epithelium and imbalance in sex steroid signaling. *PLoS One* 7:e38457. 10.1371/journal.pone.0038457 22701646 PMC3368854

[B19] Blom E. (1973). The ultrastructure of some characteristic sperm defects and a proposal for a new classification of the bull spermiogram. *Nord. Vet. Med*. 25 383–391. 4768226

[B20] Boerke A. Dieleman S. J. Gadella B. M. (2007). A possible role for sperm RNA in early embryo development. *Theriogenology* 68(Suppl. 1) S147–S155. 10.1016/j.theriogenology.2007.05.058 17583784

[B21] Bouhallier F. Allioli N. Lavial F. Chalmel F. Perrard M.-H. Durand P. (2010). Role of miR-34c microRNA in the late steps of spermatogenesis. *RNA* 16 720–731. 10.1261/rna.1963810 20150330 PMC2844620

[B22] Caballero J. Frenette G. Amours O. Belleannée C. Lacroix-Pepin N. Robert C. (2012). Bovine sperm raft membrane associated Glioma Pathogenesis-Related 1-like protein 1 (GliPr1L1) is modified during the epididymal transit and is potentially involved in sperm binding to the zona pellucida. *J. Cell. Physiol.* 227 3876–3886. 10.1002/jcp.24099 22552861

[B23] Capra E. Turri F. Lazzari B. Cremonesi P. Gliozzi T. M. Fojadelli I. (2017). Small RNA sequencing of cryopreserved semen from single bull revealed altered miRNAs and piRNAs expression between High- and Low-motile sperm populations. *BMC Genomics* 18:14. 10.1186/s12864-016-3394-7 28052756 PMC5209821

[B24] Celeghini E. C. C. Alves M. B. R. de Arruda R. P. de Rezende G. M. Florez-Rodriguez S. A. de Sá Filho M. F. (2019). Efficiency of CellROX deep red ^®^ and CellROX orange ^®^ fluorescent probes in identifying reactive oxygen species in sperm samples from high and low fertility bulls. *Anim. Biotechnol.* [Epub ahead of print]. 10.1080/10495398.2019.1654485 31424334

[B25] Celeghini E. C. C. de Arruda R. P. de Andrade A. F. C. Nascimento J. Raphael C. F. (2007). Practical techniques for bovine sperm simultaneous fluorimetric assessment of plasma, acrosomal and mitochondrial membranes. *Reprod. Domest. Anim.* 42 479–488. 10.1111/j.1439-0531.2006.00810.x 17845603

[B26] Comazzetto S. Di Giacomo M. Rasmussen K. D. Much C. Azzi C. Perlas E. (2014). Oligoasthenoteratozoospermia and Infertility in Mice Deficient for miR-34b/c and miR-449 Loci. *PLoS Genet.* 10:e1004597. 10.1371/journal.pgen.1004597 25329700 PMC4199480

[B27] Conine C. C. Sun F. Song L. Rivera-Pérez J. A. Rando O. J. (2018). Small RNAs gained during epididymal transit of sperm are essential for embryonic development in mice. *Dev. Cell* 46 470.e3–480.e3. 10.1016/j.devcel.2018.06.024 30057276 PMC6103825

[B28] Conine C. C. Sun F. Song L. Rivera-Pérez J. A. Rando O. J. (2019). MicroRNAs absent in caput sperm are required for normal embryonic development. *Dev. Cell* 50 7–8. 10.1016/j.devcel.2019.06.007 31265813

[B29] Cooper T. G. Yeung C.-H. (2003). Acquisition of volume regulatory response of sperm upon maturation in the epididymis and the role of the cytoplasmic droplet. *Microsc. Res. Tech.* 61 28–38. 10.1002/jemt.10314 12672120

[B30] Correa J. R. Pace M. M. Zavos P. M. (1997). Relationships among frozen-thawed sperm characteristics assessed via the routine semen analysis, sperm functional tests and fertility of bulls in an artificial insemination program. *Theriogenology* 48 721–731. 10.1016/S0093-691X(97)00296-3 16728166

[B31] Cui L. Fang L. Shi B. Qiu S. Ye Y. (2015). Spermatozoa micro ribonucleic acid–34c level is correlated with intracytoplasmic sperm injection outcomes. *Fertil. Steril.* 104 312.e1–317.e1. 10.1016/j.fertnstert.2015.05.003 26051092

[B32] Curry E. Safranski T. J. Pratt S. L. (2011). Differential expression of porcine sperm microRNAs and their association with sperm morphology and motility. *Theriogenology* 76 1532–1539. 10.1016/j.theriogenology.2011.06.025 21872314

[B33] D’Amours O. Frenette G. Bordeleau L.-J. Allard N. Leclerc P. Blondin P. (2012). Epididymosomes transfer epididymal sperm binding protein 1 (ELSPBP1) to dead spermatozoa during epididymal transit in bovine. *Biol. Reprod.* 87 1–11. 10.1095/biolreprod.112.100990 22875906

[B34] Evenson D. P. Wixon R. (2006). Clinical aspects of sperm DNA fragmentation detection and male infertility. *Theriogenology* 65 979–991. 10.1016/j.theriogenology.2005.09.011 16242181

[B35] Fagerlind M. Stalhammar H. Olsson B. Klinga-Levan K. (2015). Expression of miRNAs in bull spermatozoa correlates with fertility rates. *Reprod. Domest. Anim.* 50 587–594. 10.1111/rda.12531 25998690

[B36] Farrell P. B. Presicce G. A. Brockett C. C. Foote R. H. (1998). Quantification of bull sperm characteristics measured by computer-assisted sperm analysis (CASA) and the relationship to fertility. *Theriogenology* 49 871–879. 10.1016/S0093-691X(98)00036-3 10732095

[B37] Feugang J. M. Rodriguez-Osorio N. Kaya A. Wang H. Page G. Ostermeier G. C. (2010). Transcriptome analysis of bull spermatozoa: implications for male fertility. *Reprod. Biomed. Online* 21 312–324. 10.1016/j.rbmo.2010.06.022 20638337

[B38] Fishman E. L. Jo K. Nguyen Q. P. H. Kong D. Royfman R. Cekic A. R. (2018). A novel atypical sperm centriole is functional during human fertilization. *Nat. Commun.* 9:2210. 10.1038/s41467-018-04678-8 29880810 PMC5992222

[B39] Gadea J. Parrington J. Kashir J. Coward K. (2013). “The male reproductive tract and spermatogenesis,” in *Textbook of Clinical Embryology*, eds Coward K. Wells D. (Cambridge: Cambridge University Press), 18–26. 10.1017/cbo9781139192736.005

[B40] Gillan L. Evans G. Maxwell W. M. C. (2005). Flow cytometric evaluation of sperm parameters in relation to fertility potential. *Theriogenology* 63 445–457. 10.1016/j.theriogenology.2004.09.024 15626410

[B41] Gillan L. Kroetsch T. Chis Maxwell W. M. Evans G. (2008). Assessment of in vitro sperm characteristics in relation to fertility in dairy bulls. *Anim. Reprod. Sci.* 103 201–214. 10.1016/j.anireprosci.2006.12.010 17208395

[B42] Gyllensten U. Wharton D. Josefsson A. Wilson A. C. (1991). Paternal inheritance of mitochondrial DNA in mice. *Nature* 352 255–257. 10.1038/352255a0 1857422

[B43] Hayashi K. Chuva de Sousa Lopes S. M. Kaneda M. Tang F. Hajkova P. Lao K. (2008). MicroRNA biogenesis is required for mouse primordial germ cell development and spermatogenesis. *PLoS One* 3:e0001738. 10.1371/journal.pone.0001738 18320056 PMC2254191

[B44] Hecht N. B. Liem H. Kleene K. C. Distel R. J. Ho S. (1984). Maternal inheritance of the mouse mitochondrial genome is not mediated by a loss or gross alteration of the paternal mitochondrial DNA or by methylation of the oocyte mitochondrial DNA. *Dev. Biol.* 102 452–461. 10.1016/0012-1606(84)90210-0 6323235

[B45] Hermo L. Oka R. Morales C. R. (1994). Secretion and endocytosis in the male reproductive tract: a role in sperm maturation. *Int. Rev. Cytol.* 154 106–189. 10.1016/S0074-7696(08)62199-3 8083031

[B46] Hirohashi N. Yanagimachi R. (2018). Sperm acrosome reaction: its site and role in fertilization. *Biol. Reprod.* 99 127–133. 10.1093/biolre/ioy045 29462288

[B47] Inaba K. (2003). Molecular architecture of the sperm flagella: molecules for motility and signaling. *Zool. Sci.* 20 1043–1056. 10.2108/zsj.20.1043 14578564

[B48] Irvine D. S. (1998). Epidemiology and aetiology of male infertility. *Hum. Reprod.* 13 33–38. 10.1093/humrep/13.1.33 9663768

[B49] Jerczynski O. Lacroix-Pépin N. Boilard E. Calvo E. Bernet A. Fortier M. A. (2016). Role of Dicer1-dependent factors in the paracrine regulation of epididymal gene expression. *PLoS One* 11:e0163876. 10.1371/journal.pone.0163876 27695046 PMC5047620

[B50] Jodar M. Selvaraju S. Sendler E. Diamond M. P. Krawetz S. A. (2013). The presence, role and clinical use of spermatozoal RNAs. *Hum. Reprod. Update* 19 604–624. 10.1093/humupd/dmt031 23856356 PMC3796946

[B51] Kashir J. Heindryckx B. Jones C. De Sutter P. Parrington J. Coward K. (2010). Oocyte activation, phospholipase C zeta and human infertility. *Hum. Reprod. Update* 16 690–703. 10.1093/humupd/dmq018 20573804

[B52] Kasimanickam V. Kasimanickam R. Arangasamy A. Saberivand A. Stevenson J. S. Kastelic J. P. (2012). Association between mRNA abundance of functional sperm function proteins and fertility of Holstein bulls. *Theriogenology* 78 2007–2019. 10.1016/j.theriogenology.2012.07.016 23040061

[B53] Kotaja N. (2014). MicroRNAs and spermatogenesis. *Fertil. Steril.* 101 1552–1562. 10.1016/j.fertnstert.2014.04.025 24882619

[B54] Krawetz S. A. (2005). Paternal contribution: new insights and future challenges. *Nat. Rev. Genet.* 6 633–642. 10.1038/nrg1654 16136654

[B55] Kwon W.-S. Rahman M. Lee J.-S. Kim J. Yoon S.-J. Park Y.-J. (2014). A comprehensive proteomic approach to identifying capacitation related proteins in boar spermatozoa. *BMC Genomics* 15:897. 10.1186/1471-2164-15-897 25315394 PMC4287242

[B56] Kwon W.-S. Rahman M. S. Lee J.-S. You Y.-A. Pang M.-G. (2015). Improving litter size by boar spermatozoa: application of combined H33258/CTC staining in field trial with artificial insemination. *Andrology* 3 552–557. 10.1111/andr.12020 25767078

[B57] Li Y. Li R. H. Ran M. X. Zhang Y. Liang K. Ren Y. N. (2018). High throughput small RNA and transcriptome sequencing reveal capacitation-related microRNAs and mRNA in boar sperm. *BMC Genomics* 19:1–12. 10.1186/s12864-018-5132-9 30305024 PMC6180635

[B58] Liu W. Pang R. T. K. Chiu P. C. N. Wong B. P. C. Lao K. Lee K. (2012). Sperm-borne microRNA-34c is required for the first cleavage division in mouse. *Proc. Natl. Acad. Sci. U.S.A.* 109 490–494. 10.1073/pnas.1110368109 22203953 PMC3258645

[B59] Luo S. Valencia C. A. Zhang J. Lee N.-C. Slone J. Gui B. (2018). Biparental Inheritance of Mitochondrial DNA in Humans. *Proc. Natl. Acad. Sci. U.S.A.* 115 13039–13044. 10.1073/pnas.1810946115 30478036 PMC6304937

[B60] Mattick J. S. (2001). Non-coding RNAs: the architects of eukaryotic complexity. *EMBO Rep.* 2 986–991. 10.1093/embo-reports/kve230 11713189 PMC1084129

[B61] Menezes E. S. B. Badial P. R. El Debaky H. Husna A. U. Ugur M. R. Kaya A. (2019). Sperm miR-15a and miR-29b are associated with bull fertility. *Andrologia* 52:e13412. 10.1111/and.13412 31671225

[B62] Morrell J. M. Valeanu A. S. Lundeheim N. Johannisson A. (2018). Sperm quality in frozen beef and dairy bull semen. *Acta Vet. Scand.* 60 1–10. 10.1186/s13028-018-0396-2 29973236 PMC6031104

[B63] Navara C. S. Hewitson L. C. Simerly C. R. Sutovsky P. Schatten G. (1997). The implications of a paternally derived centrosome during human fertilization: consequences for reproduction and the treatment of male factor infertility. *Am. J. Reprod. Immunol.* 37 39–49. 10.1111/j.1600-0897.1997.tb00191.x 9138452

[B64] Nixon B. Stanger S. J. Mihalas B. P. Reilly J. N. Anderson A. L. Tyagi S. (2015). The MicroRNA signature of mouse spermatozoa is substantially modified during epididymal maturation. *Biol. Reprod.* 93 1–20. 10.1095/biolreprod.115.132209 26333995

[B65] Odia M. Swanson G. Krawetz S. A. (2018). A history of why fathers’ RNA matters. *Biol. Reprod.* 99 147–159. 10.1093/biolre/ioy007 29514212

[B66] Oliveira B. M. Arruda R. P. Thomé H. E. Maturana Filho M. Oliveira G. Guimarães C. (2014). Fertility and uterine hemodynamic in cows after artificial insemination with semen assessed by fluorescent probes. *Theriogenology* 82 767–772. 10.1016/j.theriogenology.2014.06.007 25023296

[B67] Ostermeier G. C. Goodrich R. J. Diamond M. P. Dix D. J. Krawetz S. A. (2005). Toward using stable spermatozoal RNAs for prognostic assessment of male factor fertility. *Fertil. Steril.* 83 1687–1694. 10.1016/j.fertnstert.2004.12.046 15950637

[B68] Ostermeier G. C. Miller D. Huntriss J. D. Diamond M. P. Krawetz S. A. (2004). Reproductive biology: delivering spermatozoan RNA to the oocyte. *Nature* 429:2603. 10.1038/nature02602 15141202

[B69] Papaioannou M. D. Pitetti J. L. Ro S. Park C. Aubry F. Schaad O. (2009). Sertoli cell Dicer is essential for spermatogenesis in mice. *Dev. Biol.* 326 250–259. 10.1016/j.ydbio.2008.11.011 19071104 PMC2705812

[B70] Paul J. Duerksen J. D. (1975). Chromatin-associated RNA content of heterochromatin and euchromatin. *Mol. Cell. Biochem.* 9 9–16. 10.1007/BF01731728 1186664

[B71] Piomboni P. Focarelli R. Stendardi A. Ferramosca A. Zara V. (2012). The role of mitochondria in energy production for human sperm motility. *Int. J. Androl.* 35 109–124. 10.1111/j.1365-2605.2011.01218.x 21950496

[B72] Ran M. Weng B. Cao R. Li Z. Peng F. Luo H. (2018). miR-26a inhibits proliferation and promotes apoptosis in porcine immature Sertoli cells by targeting the PAK2 gene. *Reprod. Domest. Anim.* 53 1375–1385. 10.1111/rda.13254 30024056

[B73] Raposo G. Stoorvogel W. (2013). Extracellular vesicles: exosomes, microvesicles, and friends. *J. Cell Biol.* 200 373–383. 10.1083/jcb.201211138 23420871 PMC3575529

[B74] Reilly J. N. McLaughlin E. A. Stanger S. J. Anderson A. L. Hutcheon K. Church K. (2016). Characterisation of mouse epididymosomes reveals a complex profile of microRNAs and a potential mechanism for modification of the sperm epigenome. *Sci. Rep.* 6 1–15. 10.1038/srep31794 27549865 PMC4994100

[B75] Rodgers A. B. Morgan C. P. Bronson S. L. Revello S. Bale T. L. (2013). Paternal stress exposure alters sperm MicroRNA content and reprograms offspring HPA stress axis regulation. *J. Neurosci.* 33 9003–9012. 10.1523/JNEUROSCI.0914-13.2013 23699511 PMC3712504

[B76] Rodgers A. B. Morgan C. P. Leu N. A. Bale T. L. (2015). Transgenerational epigenetic programming via sperm microRNA recapitulates effects of paternal stress. *Proc. Natl. Acad. Sci. U.S.A.* 112 13699–13704. 10.1073/pnas.1508347112 26483456 PMC4640733

[B77] Rompala G. R. Simons A. Kihle B. Homanics G. E. (2018). Paternal preconception chronic variable stress confers attenuated ethanol drinking behavior selectively to male offspring in a pre-stress environment dependent manner. *Front. Behav. Neurosci.* 12:257. 10.3389/fnbeh.2018.00257 30450042 PMC6225737

[B78] Salas-Huetos A. Blanco J. Vidal F. Godo A. Grossmann M. Pons M. C. (2015). Spermatozoa from patients with seminal alterations exhibit a differential micro-ribonucleic acid profile. *Fertil. Steril.* 104 591–601. 10.1016/j.fertnstert.2015.06.015 26143365

[B79] Salas-Huetos A. Blanco J. Vidal F. Grossmann M. Pons M. C. Garrido N. (2016). Sperm from normozoospermic fertile and infertile individuals convey a distinct miRNA cargo. *Andrology* 4 1–9. 10.1111/andr.12276 27676136

[B80] Samans B. Yang Y. Krebs S. Sarode G. V. Blum H. Reichenbach M. (2014). Uniformity of nucleosome preservation pattern in mammalian sperm and Its connection to repetitive DNA elements. *Dev. Cell* 30 23–35. 10.1016/j.devcel.2014.05.023 24998597

[B81] Sathananthan A. H. Kola I. Osborne J. Trounson A. Ng S. C. Bongso A. (1991). Centrioles in the beginning of human development. *Proc. Natl. Acad. Sci. U.S.A.* 88 4806–4810. 10.1073/pnas.88.11.4806 2052559 PMC51755

[B82] Satouh Y. Inoue N. Ikawa M. Okabe M. (2012). Visualization of the moment of mouse sperm-egg fusion and dynamic localization of IZUMO1. *J. Cell Sci.* 125 4985–4990. 10.1242/jcs.100867 22946049

[B83] Saunders C. M. Larman M. G. Parrington J. Cox L. J. Royse J. Blayney L. M. (2002). PLC ζ: a sperm-specific trigger of Ca 2 + oscillations in eggs and embryo development. *Development* 3544 3533–3544. 10.1242/dev.129.15.3533 12117804

[B84] Schatten H. Sun Q.-Y. (2009). The role of centrosomes in mammalian fertilization and its significance for ICSI. *Mol. Hum. Reprod.* 15 531–538. 10.1093/molehr/gap049 19549764 PMC2734160

[B85] Schuster A. Skinner M. K. Yan W. (2016). Ancestral vinclozolin exposure alters the epigenetic transgenerational inheritance of sperm small noncoding RNAs. *Environ. Epigenet.* 2:dvw001. 10.1093/eep/dvw001 27390623 PMC4933025

[B86] Sellem E. Broekhuijse M. L. W. J. Chevrier L. Camugli S. Schmitt E. Schibler L. (2015). Use of combinations of in vitro quality assessments to predict fertility of bovine semen. *Theriogenology* 84 1447.e5–1454.e5. 10.1016/j.theriogenology.2015.07.035 26296523

[B87] Sharma U. Conine C. C. Shea J. M. Boskovic A. Derr A. G. Bing X. Y. (2016). Biogenesis and function of tRNA fragments during sperm maturation and fertilization in mammals. *Science* 351 391–396. 10.1126/science.aad6780 26721685 PMC4888079

[B88] Sharma U. Sun F. Conine C. C. Reichholf B. Kukreja S. Herzog V. A. (2018). Small RNAs are trafficked from the epididymis to developing mammalian sperm. *Dev. Cell* 46 481–494. 10.1016/j.devcel.2018.06.023 30057273 PMC6103849

[B89] Simerly C. Manil-Ségalen M. Castro C. Hartnett C. Kong D. Verlhac M. H. (2018). Separation and loss of centrioles from primordidal germ cells to mature oocytes in the mouse. *Sci. Rep.* 8 1–17. 10.1038/s41598-018-31222-x 30143724 PMC6109097

[B90] Simerly C. Wu G.-J. Zoran S. Ord T. Rawlins R. Jones J. (1995). The paternal inheritance of the centrosome, the cell’s microtubule-organizing center, in humans, and the implications for infertility. *Nat. Med.* 1 47–52. 10.1038/nm0195-47 7584952

[B91] Sipilä P. Björkgren I. (2016). Segment-specific regulation of epididymal gene expression. *Reproduction* 152 R91–R99. 10.1530/REP-15-0533 27222594

[B92] Smorag L. Zheng Y. Nolte J. Zechner U. Engel W. Pantakani D. V. K. (2012). MicroRNA signature in various cell types of mouse spermatogenesis: evidence for stage-specifically expressed miRNA-221, -203 and -34b-5p mediated spermatogenesis regulation. *Biol. Cell* 104 677–692. 10.1111/boc.201200014 22909339

[B93] Song G. J. Lewis V. (2008). Mitochondrial DNA integrity and copy number in sperm from infertile men. *Fertil. Steril.* 90 2238–2244. 10.1016/j.fertnstert.2007.10.059 18249386

[B94] Sullivan R. (2015). Epididymosomes: a heterogeneous population of microvesicles with multiple functions in sperm maturation and storage. *Asian J. Androl.* 17 726–729. 10.4103/1008-682X.155255 26112475 PMC4577580

[B95] Sullivan R. Frenette G. Girouard J. (2007). Epididymosomes are involved in the acquisition of new sperm proteins during epididymal transit. *Asian J. Androl.* 9 483–491. 10.1111/j.1745-7262.2007.00281.x 17589785

[B96] Sullivan R. Saez F. (2013). Epididymosomes, prostasomes, and liposomes: their roles in mammalian male reproductive physiology. *Reproduction* 146 R21–35. 10.1530/REP-13-0058 23613619

[B97] Tang F. Kaneda M. O’Carroll D. Hajkova P. Barton S. C. Sun Y. A. (2007). Maternal microRNAs are essential for mouse zygotic development. *Genes Dev.* 21 644–648. 10.1101/gad.418707 17369397 PMC1820938

[B98] Tscherner A. Gilchrist G. Smith N. Blondin P. Gillis D. LaMarre J. (2014). MicroRNA-34 family expression in bovine gametes and preimplantation embryos. *Reprod. Biol. Endocrinol.* 12:85. 10.1186/1477-7827-12-85 25179211 PMC4162940

[B99] Vincent P. Underwood S. L. Dolbec C. Bouchard N. Kroetsch T. Blondin P. (2012). Bovine semen quality control in artificial insemination centers. *Anim. Reprod.* 9 153–165.

[B100] Wang C. Yang C. Chen X. Yao B. Yang C. Zhu C. (2011). Altered profile of seminal plasma microRNAs in the molecular diagnosis of male infertility. *Clin. Chem.* 57 1722–1731. 10.1373/clinchem.2011.169714 21933900

[B101] Wang M. Gao Y. Qu P. Qing S. Qiao F. Zhang Y. (2017). Sperm-borne miR-449b influences cleavage, epigenetic reprogramming and apoptosis of SCNT embryos in bovine. *Sci. Rep.* 7 1–12. 10.1038/s41598-017-13899-8 29042680 PMC5645405

[B102] Weinhold B. (2006). Epigenetics: the science of change. *Environ. Health Perspect.* 114 160–167. 10.1289/ehp.114-a160 PMC1392256 16507447

[B103] Wightman B. Ha I. Ruvkun G. (1993). Posttranscriptional regulation of the heterochronic gene lin- 14 by lin-4 mediates temporal pattern formation in *C. elegans*. *Cell* 75 855–862. 10.1016/0092-8674(93)90530-4 8252622

[B104] Wu A. T. H. Sutovsky P. Manandhar G. Xu W. Katayama M. Day B. N. (2007). PAWP, a sperm-specific WW domain-binding protein, promotes meiotic resumption and pronuclear development during fertilization. *J. Biol. Chem.* 282 12164–12175. 10.1074/jbc.M609132200 17289678

[B105] Wu J. Bao J. Kim M. Yuan S. Tang C. Zheng H. (2014). Two miRNA clusters, miR-34b/c and miR-449, are essential for normal brain development, motile ciliogenesis, and spermatogenesis. *Proc. Natl. Acad. Sci. U.S.A.* 111 E2851–E2857. 10.1073/pnas.1407777111 24982181 PMC4104921

[B106] Yuan S. Schuster A. Tang C. Yu T. Ortogero N. Bao J. (2016). Sperm-borne miRNAs and endo-siRNAs are important for fertilization and preimplantation embryonic development. *Development* 143 635–647. 10.1242/dev.131755 26718009 PMC4760322

[B107] Zegers-Hochschild F. Adamson G. D. Dyer S. Racowsky C. De Mouzon J. Sokol R. (2017). The international glossary on infertility and fertility care, 2017. *Hum. Reprod.* 32 1786–1801. 10.1093/humrep/dex234 29117321 PMC5850297

[B108] Zhou J.-H. Zhou Q.-Z. Yang J.-K. Lyu X.-M. Bian J. Guo W.-B. (2017). MicroRNA-27a-mediated repression of cysteine-rich secretory protein 2 translation in asthenoteratozoospermic patients. *Asian J. Androl.* 19:591. 10.4103/1008-682X.185001 27517483 PMC5566855

[B109] Zhou W. Stanger S. J. Anderson A. L. Bernstein I. R. De Iuliis G. N. McCluskey A. (2019). Mechanisms of tethering and cargo transfer during epididymosome-sperm interactions. *BMC Biol.* 17:1–18. 10.1186/s12915-019-0653-5 30999907 PMC6474069

[B110] Zimmermann C. Romero Y. Warnefors M. Bilican A. Borel C. Smith L. B. (2014). Germ cell-specific targeting of DICER or DGCR8 reveals a novel role for endo-siRNAs in the progression of mammalian spermatogenesis and male fertility. *PLoS One* 9:e107023. 10.1371/journal.pone.0107023 25244517 PMC4171096

[B111] Zini A. Agarwal A. (2011). *Sperm Chromatin.* New York, NY: Springer.

[B112] Ziyyat A. (2001). Differential gene expression in pre-implantation embryos from mouse oocytes injected with round spermatids or spermatozoa. *Hum. Reprod.* 16 1449–1456. 10.1093/humrep/16.7.1449 11425828

